# Medical Dictionaries

**Published:** 1897-05

**Authors:** 


MEDICAL DICTIONARIES. The improvement in medical dictionaries has been so marked during the past few years, that it is worthy of comment and praise. We especially refer to the three following :

"Gould's Illustrated Dictionary of Medicine, Biology and Allied Sciences," including Pronunciation, Accentuation, Derivation and Definition of the terms used in medicine, anatomy, surgery and all other allied sciences, is so pre-eminently superior to any that has preceded it, that it is beyond question the greatest in existenee. It may be truly said that anything not found in this great and comprehensive work is not worthy of research. The Publishers, P. Blakiston, Son & Co., of Philadelphia, deserve great credit for the manner in which they have presented the work in question. The illustrations are not only numerous but are well executed, and the author in his preface announces that he knows the blessing of a publisher and of a printer who take pride in their work above and beyond the question of dollars and cents. Happy Author, commendable Publisher and Printer.

A smaller dictionary, but at the same time an excellent one, is "Duane's Students' Dictionary of Medicine and the Allied Sciences." Publishers : Lea Brothers & Co., Philadelphia. Its contents comprise the pronunciation, derivation and full explanation of medical terms, together with much collateral descriptive matter, numerous tables, etc.

"A Pocket Dictionary of 21,000 Medical Words," pronounced and defined is another of Dr. George M. Gould's works, which is also published in an convenient and attractive style by P. Blakiston, Son & Co., Philadelphia. It is both useful and convenient.

